# A malaria vaccine candidate based on an epitope of the *Plasmodium falciparum* RH5 protein

**DOI:** 10.1186/1475-2875-13-326

**Published:** 2014-08-18

**Authors:** Rosalynn L Ord, Jerri C Caldeira, Marilis Rodriguez, Amy Noe, Bryce Chackerian, David S Peabody, Gabriel Gutierrez, Cheryl A Lobo

**Affiliations:** Department of Blood-Borne Parasites, New York Blood Center, New York, NY 10065 USA; Department of Molecular Genetics and Microbiology, University of New Mexico School of Medicine, Albuquerque, New Mexico USA; Leidos, Inc, Reston, Viginia USA

## Abstract

**Background:**

The *Plasmodium falciparum* protein RH5 is an adhesin molecule essential for parasite invasion of erythrocytes. Recent studies show that anti-PfRH5 sera have potent invasion-inhibiting activities, supporting the idea that the PfRH5 antigen could form the basis of a vaccine. Therefore, epitopes recognized by neutralizing anti-PfRH5 antibodies could themselves be effective vaccine immunogens if presented in a sufficiently immunogenic fashion. However, the exact regions within PfRH5 that are targets of this invasion-inhibitory activity have yet to be identified.

**Methods:**

A battery of anti-RH5 monoclonal antibodies (mAbs) were produced and screened for their potency by inhibition of invasion assays *in vitro*. Using an anti-RH5 mAb that completely inhibited invasion as the selecting mAb, affinity-selection using random sequence peptide libraries displayed on virus-like particles of bacteriophage MS2 (MS2 VLPs) was performed. VLPs were sequenced to identify the specific peptide epitopes they encoded and used to raise specific antisera that was in turn tested for inhibition of invasion.

**Results:**

Three anti-RH5 monoclonals (0.1 mg/mL) were able to inhibit invasion *in vitro* by >95%. Affinity-selection with one of these mAbs yielded a VLP which yielded a peptide whose sequence is identical to a portion of PfRH5 itself. The VLP displaying the peptide binds strongly to the antibody, and in immunized animals elicits an anti-PfRH5 antibody response. The resulting antisera against the specific VLP inhibit parasite invasion of erythrocytes more than 90% *in vitro*.

**Conclusions:**

Here, data is presented from an anti-PfRH5 mAb that completely inhibits erythrocyte invasion by parasites *in vitro*, one of the few anti-malarial monoclonal antibodies reported to date that completely inhibits invasion with such potency, adding to other studies that highlight the potential of PfRH5 as a vaccine antigen. The specific neutralization sensitive epitope within RH5 has been identified, and antibodies against this epitope also elicit high anti-invasion activity, suggesting this epitope could form the basis of an effective vaccine against malaria.

## Background

Clinical symptoms of malaria are due to the blood-stage of infection in which merozoites invade erythrocytes, and multiply until the cell bursts, thereby liberating progeny merozoites that in turn invade new erythrocytes [1]. The parasite invades erythrocytes via multiple pathways. In the case of *Plasmodium falciparum* the two principal routes are the sialic acid (SA)-dependent and SA-independent pathways [2]. The glycophorin (GP) receptors are the main sialylated proteins on the RBC surface, and the parasite adhesins that bind GPs govern the SA-dependent pathway. They include members of the erythrocyte binding ligand (EBL) family, such as EBA-175 [1, 3–9], and PfRH1 [10]. Antigens identified as utilizing the SA-independent pathway are mainly, but not exclusively, comprised of the reticulocyte binding protein-like homologues (RH), RH2a, RH2b, RH4 and RH5 [9, 11–13]. The RBC receptors bound by RH2a and RH2b have not yet been fully identified; RH4 binds to complement receptor (CR) 1 [14], and RH5 binds to basigin [15].

PfRH5 appears to be essential for erythrocyte invasion. Not only is the PfRH5-basigin interaction required for erythrocyte invasion by all tested strains of *P. falciparum* [15], but repeated unsuccessful attempts to delete the PfRH5 gene suggest the protein is needed for viability [12, 16, 17]. Whole genome sequencing of almost 300 clinical *P. falciparum* isolates identified only five non-synonymous PfRH5 SNPs [18], revealing that the protein has limited sequence polymorphism. Further, our lab and others have shown potent inhibition of invasion using antibodies raised against recombinant PfRH5 protein [19–21]. Recently, naturally acquired anti-PfRH5 antibodies from the sera of malaria patients were also shown to be inhibitory *in vitro* and correlate with protection from malaria [22, 23]. All this points to PfRH5’s promise as a vaccine antigen, and to the possibility that epitopes recognized by neutralizing antibodies could themselves be effective vaccine immunogens if presented in a sufficiently immunogenic fashion. However, until recently [24] no study has identified the actual regions on PfRH5 that are either responsible for red cell binding or are targets of this invasion-inhibitory activity.

In this study, a monoclonal antibody that completely prevents red cell invasion by the parasite *in vitro* was identified, as well as two other mAbs that inhibit invasion by greater than 95%, of any anti-malarial monoclonal antibodies that are able to so potently inhibit parasite invasion. Using a bacteriophage virus-like particle (VLP) based peptide display platform, the specific neutralization-sensitive epitope targeted by one of these monoclonal antibodies was identified. Vaccination with VLPs displaying this epitope elicits antibodies that, in turn, potently inhibit erythrocyte invasion by *P. falciparum*.

## Methods

### Animal work and ethics statement

Animal protocols in this study were reviewed and approved by the A&G (protocol #AG-01) and University of New Mexico (protocol #12-100865-HSC) Institutional Animal Care and Use Committees (IACUC) to ensure they met with strict accordance to the recommendations of the Guide for the Care and Use of Laboratory Animals of the NIH. Isoflurane was used to sedate the mice for immunizations, and all efforts were made to minimize suffering at all times.

### Preparation of mouse hybridomas and monoclonal antibodies

Monoclonal antibodies were generated by Precision Antibody (a wholly owned service division of A&G Pharmaceutical, Inc.) using their proprietary custom monoclonal antibody development service. SJL/J mice (derived from Swiss Webster) were immunized with recombinant full-length wheat germ PfRH5 using Precision Antibody’s proprietary protocol and adjuvant. Immunogenicity in mice was assessed based on endpoint ELISA using recombinant full-length wheat germ PfRH5 (100 ng/well). Once endpoint titers greater than 1:50,000 were reached, splenocytes from the mouse with the highest titer were harvested for fusion. Hybridomas were single-cell cloned and culture supernatants tested for activity via ELISA using recombinant full-length wheat germ PfRH5 (100 ng/well). Crude supernatants from the highest responders were harvested for testing in invasion inhibition assays. Supernatants which inhibited parasite invasion by ≥60% were selected for monoclonal purification.

### Invasion inhibition assay (IIA)

Parasite culture maintenance and IIAs were performed as described [20]. Purified IgG from naïve mouse sera was used at equivalent concentrations as negative controls in all IIAs. Crude supernatants from mouse hybridoma cultures were tested at 1:5 dilutions. Purified monoclonal antibodies were assayed at 0.025 to 0.1 mg/mL. Cardiac bleed sera from the four animals immunized with the 5A08-VLP were pooled and the IgG fraction was purified using Protein G Sepharose beads, dialyzed overnight in 1× PBS. Concentrations of IgG were determined against a BSA standard curve on a spectrophotometer (BioRad) and IIAs performed at 0.1 mg/mL to 1 mg/mL in 3D7. All IIAs were done at least 2–3 times, in triplicate.

### Immunofluorescence assay (IFA)

Mature schizont stage 3D7 parasites were smeared onto slides and stored at -70°C before use. Slides were thawed and fixed with 10% methanol/90% acetone for 20 min at room temperature. After air-drying, the smears were coated with anti-PfRH5 monoclonal antibody (1:20 in 1× PBS/1% BSA) and incubated at room temperature for 1 h. Slides were washed by shaking in 1× PBS for 5 min then incubated with FITC-conjugated anti-mouse antibody (1:50 in 1× PBS/1% BSA) for 1 h at room temperature protected from light. All slides were washed by shaking in 1x PBS for 5 min then mounted using 10 μg/mL DAPI. Slides were observed under UV light. Supernatants which recognized native PfRH5 antigen by IFA were selected for monoclonal purification. Slides with mature 3D7 parasites were fixed and air dried as before and co-stained with mouse anti-5A08-VLP (“Mα5A08-VLP”; 1:20) and rabbit anti-PfRhop148 [25] (“RαPfRhop148”; 1:100) or with mouse anti-5A08-VLP (“Mα5A08-VLP”; 1:20) and anti-RhopH3 [25] (RαRhopH3; 1:500) in 1× PBS/1% BSA. All slides were washed by shaking in 1× PBS for 5 min then incubated with a mixture of FITC-conjugated anti-mouse antibody (1:50) and TRITC-conjugated anti-rabbit IgG (1:50) in 1× PBS/1% BSA) for 1 h at room temperature protected from light. All slides were washed by shaking in 1× PBS for 5 min.

### Immunoblotting

Saponin-lysed pellets from mature-stage parasites, was used to make a native protein lysate from mature 3D7 parasites. This was separated on 10% SDS-PAGE gels by electrophoresis, transferred to nylon membrane. Immunoblotting of the membranes was performed using standard techniques with purified monoclonal antibody at 1:2 dilution, or polyclonal anti-VLP sera at 1:400 dilution, as the primary antibodies. Mouse anti-HRP at 1:3,000 dilution was used the secondary antibody.

### VLP libraries and affinity selection

Three rounds of affinity-selection were conducted by biopanning using an equal a mixture of four different random sequence peptide libraries constructed by methods described previously [26]. Each library displayed 6mer, 7mer, 8mer, or 10mer peptides, was constructed independently, and contained about 10^10^ individual members.

### 5A08-VLP immunizations

The 5A08-VLP selectant was purified as described [27] and three mice were immunized three times by intramuscular injection with 5 μg at two-week intervals in the presence of the GLA-SE adjuvant. Two weeks after the last immunization the animals were sacrificed and their sera collected.

### ELISA

The 5A08-VLP selectant was purified by chromatography on Sepharose CL4B [27] and tested for its ability to bind 2E11, 5A08, and 5A03 in direct ELISA. Purified VLPs (500 ng) were adsorbed to a 96-well flat-bottomed ELISA plate (Immulon 2) overnight at 4°C. Plates were blocked with 5% (w/v) BSA in PBS for 2 h at 37°C and then washed three times with 1x PBS. 1uL of sera was diluted in 50 μL of PBS containing 2% BSA was added to the plates and incubated for 1 h at 37°C. After three washes with 1× PBS, an HRP-conjugated goat anti-mouse IgG secondary antibody (Sigma, diluted 1:5,000) was added and incubated for 1 h at 37°C. The plates were washed again three times with 1x PBS, and the colorimetric substrate, 2,2’-azino-bis(3-ethylbenzthiazoline-6-sulphonic acid) (ABTS) was added and incubated at room temperature until adequate color development was obtained (about 15 min). Absorbance at 405 nm was measured. All reactions were performed in duplicate. Peptide ELISA was conducted by similar means, but the synthetic peptide SAIKKPVTGGGC (500 ng) was cross-linked through its C-terminal cysteine residue to the amino groups of lysines on streptavidin coated wells using the heterobifunctional cross-linker, succinimidyl 6-[(β-maleimidopropionamido)hexanoate] (SMPH). After washing, the peptide was reacted with the serial dilutions of sera for 2 h at room temperature. Reaction with secondary antibody and colorimetric assay were as described above. In this case reactions were conducted in triplicate.

## Results and discussion

### Screening hybridoma supernatants

**Figure 1:**
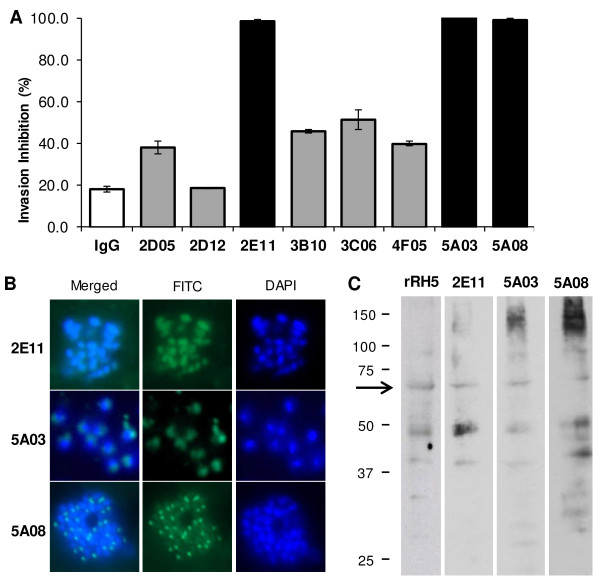
**Purified anti-PfRH5 monoclonal antibodies inhibit invasion of** ***P. falciparum*** **strain 3D7. (A)** The efficacy of purified anti-PfRH5 monoclonal antibodies (mAbs) to prevent invasion of 3D7 parasites was determined at 0.1 mg/mL. Three mAbs, 2E11, 5A03 and 5A08 were able to inhibit invasion by 98%, 100% and 99%, respectively, while the inhibition of the remaining mAbs ranged from 20-45%. Error bars show standard error. **(B)** Immunofluorescence assays show these three mAbs with high inhibition recognize native PfRH5 located at the apical end of merozoites. **(C)** Western blot assay on mature 3D7 parasite lysate with these three purified mAbs also shows that native PfRH5 is recognized by each of these antibodies.

An ideal malaria vaccine antigen would be highly conserved across a broad spectrum of *Plasmodium falciparum* strains, and would be essential to parasite viability and reproduction so resistance could not be easily acquired by mutation, or by simply switching off expression. The merozoite protein PfRH5 seems to meet these criteria, especially as several attempts to delete the PfRH5 gene have been unsuccessful, suggesting that PfRH5 is probably essential to parasite viability [12, 16, 17]. To identify epitopes recognized by invasion-inhibiting antibodies, we first identified potent neutralizing monoclonal antibodies by screening 35 hybridoma supernatants from mice immunized with full-length recombinant PfRH5 [20] for the ability to inhibit invasion of erythrocytes by *P. falciparum* strain 3D7 in the invasion inhibition assay (IIA) and to recognize native PfRH5 antigen by immunofluorescence (IFA). Supernatants which showed inhibition ≥60% or were positive by IFA were down selected for purification. Of the eight purified monoclonal antibodies produced, three inhibited 3D7 invasion by >95% at 0.1 mg/ml (mAbs 2E11, 5A03 and 5A08; Figure 1A). The specificity of these three mAbs towards native PfRH5 antigen was confirmed by IFA and immunoblot analysis (Figures 1B and C). There have been very few antibodies reported to date that are able to inhibit invasion in vitro by such a degree and this suggests that these mAbs target the neutralizing epitope(s) of RH5.

### Affinity selection of a peptide that binds the 5A08 antibody

**Figure 2:**
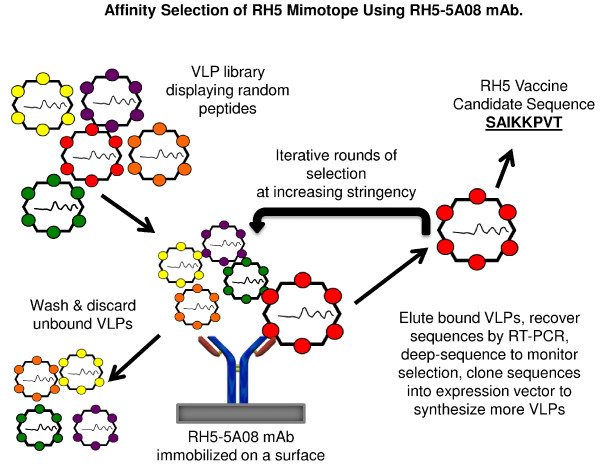
**Library construction and affinity selection of MS2 VLPs.** When expressed from a plasmid in bacteria, the coat protein of bacteriophage MS2 forms a virus-like particle (VLP), which we have adapted for peptide display and affinity-selection. A complex library of random peptide sequences is constructed at the level of plasmid DNA. When the DNA is expressed in *E. coli*, a library of corresponding VLPs is produced, as the recombinant coat proteins self-assemble into VLPs, each of which displays a different guest peptide on its surface and encapsidates its own mRNA. These VLPs are subjected to affinity selection with a specific mAb. Unbound VLPs are washed and discarded. VLPs which display a peptide with an affinity for the mAb used are eluted, and subjected to RT-PCR to generate cDNA for another round of affinity selection. After affinity selection, the VLPs were characterized to determine the sequence of the peptide.

To identify the epitope targeted specifically by the 5A08 mAb, (chosen as it completely inhibited invasion), affinity selection using random sequence peptide libraries displayed on virus-like particles of bacteriophage MS2 (MS2 VLPs) with 5A08 as the selecting mAb (Figure 2) was performed. The method is highly analogous to conventional phage display, but is based on the VLPs that form when MS2 coat protein is expressed from a plasmid in *Escherichia coli*. The ability to display libraries of diverse peptides on the MS2 VLP and to then affinity select those rare peptides that bind a target antibody depends on two essential conditions: First, it is necessary to identify a surface-exposed site in coat protein that tolerates peptide insertions without disruption of protein folding or VLP assembly. Coat protein’s AB-loop is prominently exposed on the VLP surface, but in the wild-type protein AB-loop insertions nearly always interfere with correct folding. Fortunately, a simple means of conferring insertion tolerance was found. Coat protein is a symmetric dimer of identical polypeptide chains with the N-terminus of one monomer lying in close physical proximity to the C-terminus of the other. By duplicating the coat coding sequence and fusing the two copies into a single reading frame, a “single-chain dimer” that is dramatically more stable thermodynamically, and highly tolerant of insertions in one of its two AB-loops was created [27–29]. Second, to accomplish the linkage of phenotype to genotype that forms the basis of all such technologies, the VLP must encapsidate the nucleic acid that encodes coat protein and its guest peptide. As it happens, the MS2 VLP efficiently encapsidates its own mRNA [27], meaning that affinity selected sequences can be recovered and amplified by reverse transcription and polymerase chain reaction. Because of their multivalent presentation on the VLP surface foreign peptides are highly immunogenic. Therefore, the MS2 VLP can integrate the epitope identification and immunization functions into a single platform and the cloned products of affinity selection can be produced in bacteria, purified and then used directly as vaccines.

The plasmid vectors and methods that facilitate the construction of peptide libraries have been described [26–28]. Random sequences of varying lengths are inserted into one AB-loop of the single-chain dimer. When expressed in bacteria the recombinant coat proteins self-assemble into VLPs, each of which displays a different guest peptide on its surface and encapsidates its own mRNA In the present study, a mixture of random sequence 6-mer, 7-mer, 8-mer and 10-mer libraries, each comprised of about 10^10^ independent clones were used [27]. After bio-panning on the 5A08 target, affinity-selected sequences were recovered by reverse transcription of the coat protein-specific mRNA they contained, followed by polymerase chain reaction. The selected sequences were then re-cloned to produce VLPs for additional rounds of selection. This process normally requires several iterative selection rounds to obtain a relatively simple population of peptides that tightly bind the target antibody.

**Figure 3:**
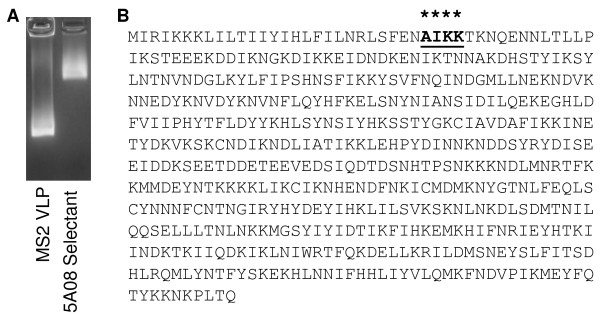
**Affinity selection using the anti-PfRH5 mAb 5A08 identifies a single, short linear epitope, AIKK. (A)** Agarose gel of purified, unmodified, MS2 VLP (left lane) and a single clone representative of the highly homogenous selectant population from twelve VLPs selected by the anti-PfRH5-5A08 mAb (right lane), stained with ethidium bromide by virtue of the RNA within each VLP. **(B)** Characterization by sequencing of clones from each round of affinity-selection showed each RH5-5A08 VLP displayed a four-amino acid sequence at its core, AIKK/R, and that by round two, only VLPs with the AIKK epitope were selected for. The full length PfRH5 sequence (from PlasmoDB, gene ID PF3D7_0424100) contains a 4-amino acid identity to the AIKK sequence that occurs only once near the PfRH5 N-terminus (amino acids 28–31, indicated with asterisks), highlighting the potential importance of this epitope with binding to the RBC receptor during parasite invasion.

In this case, however, after only two rounds a virtually homogeneous selectant population was obtained, in which each of twelve individually characterized clones displayed the peptide 8-mer, SAIKKPVT. A third round of affinity-selection also identified the SAIKKPVT sequence in 7 of 8 clones, and the very similar 8-mer TAIKKVPT was identified in one clone. Figure 3A shows the electrophoretic behaviour of the SAIKKPVT-containing particle at the second round of selection compared to unmodified MS2 VLPs. It is unlikely the selection passed through an artificial bottleneck that restricted the diversity of the selected sequence for reasons unrelated to its affinity for the antibody as independent selections gave essentially identical results, but at least one of the sequenced clones shows a slight deviation for the major sequence, replacing the serine at amino acid 1 with threonine. Also, selections were conducted in parallel on two different anti-HCV mAbs, which effectively serve as positive controls for the selection process as a whole, and at round 3 these anti-HCV selections yielded diverse peptide families, all of which show homology to their known epitopes (data not shown), indicating that the selections functioned normally.

The AIKK sequence contained within the peptide is identical to one encountered near the PfRH5 N-terminus (Figure 3B), suggesting this site represents the 5A08 epitope. It is believed that the N-terminal 21 amino acids of PfRH5 contain a signal sequence that is proteolytically removed from the mature protein [12, 13, 17]. Not only is AIKK the only sequence that survived two selection rounds, but deep sequence analysis of the 200 most abundant first round selectants (from ~2,500) shows that it is only one member of a much larger family sequence family whose common feature is the AIKK (or AIKR) tetrapeptide, and the AIK(K/R) motif always occupies amino acids 2–5 (from the N-end of the sequences characterized. The fact that by round two of affinity-selection SAIKKPVT was so clearly favored over the other members of this family suggests that amino acid residues outside the AIKK identity may serve to most effectively present the core four-amino acid epitope to the antibody in the context of the display site on the MS2 coat protein AB-loop.

Surveys of gene polymorphism by deep sequence analysis of nearly 300 different African malaria samples show the PfRH5 protein sequence is highly conserved [18, 30] and that none of the known polymorphisms affects the AIKK epitope. The specific role of the AIKK epitope in PfRH5 function is not known, but it is tempting to think the epitope participates directly in interactions with the basigin receptor on erythrocytes, and that the presence of the 5A08 monoclonal antibody directly interferes with binding. A recent report described neutralizing mAbs that bind epitopes found in at least two defined regions of the PfRH5 primary sequence [24]. Neither overlaps the predicted 5A08 epitope. Interestingly the same report found two classes of mAbs that had a severe inhibitory effect on parasite invasion, yet only one group inhibited PfRH5-Basigin binding [24].

### Immunization with the 5A08 VLP selectant elicits antibodies that recognize PfRH5 and strongly inhibit parasite entry into erythrocytes

**Figure 4:**
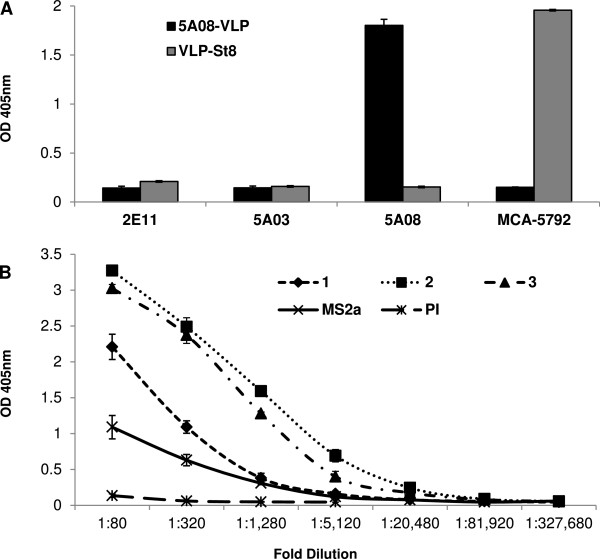
**The anti-PfRH5 mAb 5A08 specifically recognizes the affinity-selected VLP. (A)** ELISA performed with different mAbs against with the 5A08-VLP, and a MCA-5792-VLP, show that the 5A08-VLP reacts with the anti-PfRH5 5A08 mAb, but not with the two other anti-PfRH5 mAbs, 2E11 and 5A03, nor with MCA-5792 used here as a control, which reacts well with its own selectant. MCA-5792 recognizes *Staphylococcus aureus* peptidoglycan; St8 is a VLP affinity-selected for its ability to interact with MCA-5792. **(B)** The 5A08-selected VLP elicits antibodies that recognize the synthetic SAIKKPVTGGGC peptide. ELISA assay shows the anti-peptide antibody titers of sera from mice immunized with the synthetic VLP bearing the SAIKKPVTGGGC peptide. The synthetic peptide was bound to the plates by chemical cross-linking. Pooled pre-immune sera from the immunized mice show negligible anti-VLP peptide antibody titers in naïve animals. Pooled sera from mice immunized with MS2 itself also showed some reaction with the peptide, but it was much lower and decreased quickly with dilution. However, sera from all three mice immunized (indicated as 1, 2 and 3) showed high anti-VLP peptide antibody titers, two of the three giving signals well above background levels beyond the 1:5,120 dilution point.

**Figure 5:**
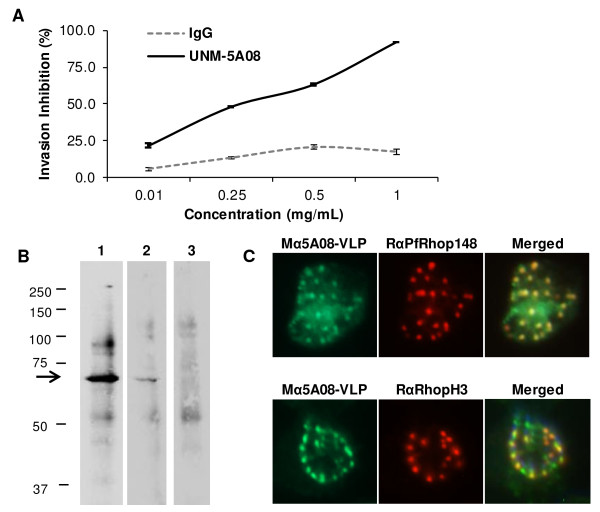
**The VLP bearing the SAIKKPVT peptide elicits antibodies that inhibit parasite invasion. (A)** Pooled sera from mice immunized with the VLP displaying the SAIKKPVT peptide were tested for their ability to inhibit 3D7 invasion (concentrations used from 0.1 mg/mL to 1.0 mg/mL). Invasion inhibition ranged from 22% to 92%. Purified IgG from naïve mouse sera was used at equivalent concentrations as negative controls. Error bars show standard error. **(B)** Immunoblotting (left panel; expected size of native PfRH5 at ~63 kDa indicated by arrow) and **(C)** immunofluorescence (right panel; apical end of merozoites are indicated by FITC staining) show the PfRH5 specific VLP sera is able to recognize native PfRH5.

The purified 5A08 VLP selectant was tested by direct ELISA for its ability to bind each of the three invasion-inhibiting anti-PfRH5 mAbs (i.e. 5A08, 5A03, and 2E11). As expected of an affinity-selectant, it bound significantly only to the 5A08 antibody (Figure 4A). Three mice were immunized with the VLP displaying the SAIKKPVT sequence by intramuscular injection with 5 μg of VLP, 3 times at two-week intervals. To assess the relative anti-peptide titers of the antisera, a synthetic peptide representing the selected sequence was synthesized and tested in ELISA for its ability to bind sera from the immunized animals using sera serially diluted from 1:80 to 1:81,920 (Figure 4B). Unsurprisingly, the 5A08-VLPs elicit antibodies that recognize a synthetic version of the immunizing peptide. Most importantly, purified IgG from the anti-5A08-VLP antiserum was potently inhibitory in GIA, showing greater than 90% inhibition of parasite entry at a concentration of 1 mg/mL IgG concentration (Figure 5A). Remembering that only a small fraction of the total IgG is likely to be specific for the 5A08 epitope, this level of inhibition promises a significantly improved route for eliciting anti-invasion antibodies. The reaction of the antisera with native PfRH5 was confirmed by Western blot analysis (Figure 5B), where it can be seen that sera against 5A08-VLP (lane 2) reacts with the same polypeptides as seen by antibodies to the full-length RH5 (lane 1). Immunoblot analysis with negative control sera directed against empty VLPs did not react with the 63 kDa PfRH5 band (lane 3). IFA analysis was also performed to confirm rhoptry localization of the target of the 5A08-VLP sera (Figure 5C). Co-staining using anti-5A08-VLP sera along with antibodies to known rhoptry markers, PfRhop148 (Figure 5C, top panel) and RhopH3 (Figure 5C, bottom panel) showed specific rhoptry localization of the target of the 5A08-VLP. These studies thus indicated that the 5A08-VLP recognizes native PfRH5 in the rhoptries.

Several groups have shown that the intact PfRH5 protein, whether administered as a recombinant protein or expressed from viral vectors, elicits antibodies that inhibit parasite entry into erythrocytes *in vitro* [18, 19, 21]. But subunit vaccines generally elicit strong responses only after several administrations, and the immunity they engender is frequently relatively short-lived [31]. This is probably because the survival potential of a plasma cell and its level of antibody production are determined during its initial interaction with antigen. Recombinant proteins, and other antigens that lack multivalency, do not strongly activate B cell activation through BCR crosslinking [32, 33]. Because of their multivalency, on the other hand, VLPs present peptide epitopes as potent immunogens that elicit strong and durable antibody responses, often after only a single administration [34]. In addition to its high immunogenicity, the MS2 VLP has the ability to identify epitopes by affinity-selection. Given a mAb with neutralizing activity, the work presented here shows that the MS2 VLP can provide a direct route to an epitope-specific vaccine candidate.

## Conclusions

These results add to other studies that point to the potential of PfRH5 as a vaccine antigen [18–24, 35], although none of the antibodies reported so far has approached the high level of invasion inhibition shown by our three mAbs, 2E11, 5A03 and 5A08, or the sera that were raised against the VLP that displays the 5A08 epitope. This highlights the relative importance of the AIKK epitope for effective parasite neutralization. Further, the work presented here shows that when displayed on the MS2 VLP even a single PfRH5 epitope can elicit antibodies that potently inhibit erythrocyte invasion by parasites. The high immunogenicity of peptides displayed multivalently on MS2 VLPs permits the use of the affinity-selected VLP itself as a vaccine immunogen. Thus the VLP integrates the epitope discovery and immunization functions into a single platform. While the PfRH5 protein itself might form the basis of an effective subunit vaccine, an epitope-specific vaccine based on the MS2 VLP platform has some advantages. Clinical trials conducted with VLP-based biologics have demonstrated that these products have good safety profiles [36–39]. Even at low doses VLPs elicit high-titer and remarkably durable antibody responses, potentially obviating the need for adjuvants [40]. Furthermore, because MS2 VLPs are produced at high levels in bacteria they should be relatively easy and cheap to manufacture, and the particle itself is relatively stable. These features are particularly important in the developing world, where the burden of malaria is the highest.
